# Degradable polyphosphoester-based aqueous two-phase systems and water-in-water emulsions

**DOI:** 10.1039/d5sc09406b

**Published:** 2026-01-09

**Authors:** Jordan A. J. McCone, Ramon ten Elshof, Niamh Bayliss, Frederik R. Wurm, Bernhard V. K. J. Schmidt

**Affiliations:** a School of Chemistry, University of Glasgow Glasgow G12 8QQ UK Bernhard.Schmidt@glasgow.ac.uk; b Sustainable Polymer Chemistry, Department of Molecules and Materials, MESA + Institute for Nanotechnology, Faculty of Science and Technology, University of Twente P.O. Box 217, 7500 AE Enschede The Netherlands Frederik.Wurm@utwente.nl

## Abstract

Completely water-based multicompartment systems have gained significant interest in polymer chemistry recently, mainly due to their interesting properties for molecular separation, material design and catalytic environments. Herein, we present new aqueous two-phase systems and water-in-water emulsions based on a polysaccharide and a polyphosphoester, namely dextran (Dex) and poly(ethylene ethyl phosphonate) (PPE), respectively. The aqueous two-phase formation is investigated *via* a phase diagram, and water-in-water emulsions are formed making use of silica nanoparticles as stabilizers. The system is designed to investigate the effect of polymer orthogonal degradation and the role of either Dex or PPE in breaking the two-phase systems or the emulsions. This development is a considerable step forward enhancing control over aqueous multi-phase systems, by making use not only of polymer components but also of their degradation to design desired properties. These properties might be a relevant avenue for developments in the research area of all-aqueous systems for encapsulation, delivery and release as well as membraneless-organelle mimics.

## Abstract 2

New aqueous two-phase systems and water-in-water emulsions are described that feature triggered degradation.

## Graphical abstract

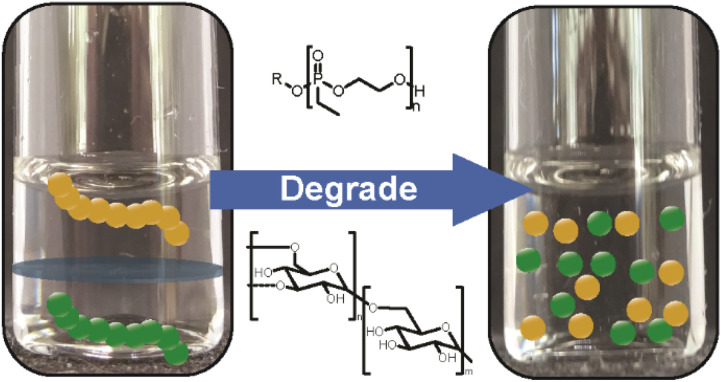

Water-soluble polymers play a crucial role in polymer science as they are found in various polymer structures,^1^*e.g.* in hydrogels,^2^ as the hydrophilic part in amphiphiles^3^ or on surfaces.^4^ These polymers find applications from every-day life like cosmetics^5^ to advanced biomedical formulations.^6^ In particular, the interaction of synthetic hydrophilic polymers with biological systems has been of high relevance, like designed hydrogel scaffolds in tissue engineering^7^ or drug delivery systems.^8^ Degradation of water-soluble polymers has received growing attention as their fate in the environment has to be controlled due to their ubiquitous use.^9,10^ Degradation could be introduced in stimulus responsive hydrophilic polymers,^11,12^ polyelectrolytes^13^ as well as cellular cryopreservation materials.^14^

In recent years, aqueous two-phase systems (ATPSs) and water-in-water (w/w) emulsions have attracted growing interest,^15^ as they are formed in mixtures of two polymers or salts in water at elevated concentration. The most frequently used example is an ATPS formed from dextran (Dex) and poly(ethylene glycol).^16^ These completely aqueous compartmentalised systems are discussed with regard to the development of artificial cells,^17^ in cell culture studies^18^ and exploited in investigation of membrane properties.^19^ Furthermore, the entirely aqueous environment in these systems has prompted discussion of various potential applications, including 3D printing,^20,21^ waste-water treatment,^22^ food,^23^ sensing^24^ or catalysis.^25^ The most common application of ATPSs is the extraction of biomacromolecules, as these molecules partition between the phases according to their affinity for the species present in each phase.^26^

Directly related to ATPSs are all aqueous w/w emulsions, which form upon dispersion of ATPSs.^27^ Compared to traditional water- and oil-based emulsions, w/w emulsions do not rely on organic solvents/oils, thereby increasing biocompatibility.^28^ Nevertheless, small molecule surfactants are generally ineffective at stabilizing w/w emulsions, as they cannot adequately cover the broad interface between both phases. Furthermore, w/w emulsions lack a hydrophobic phase that would interact with the hydrophobic segments of surfactants. Therefore, stabilization of w/w emulsions is usually achieved using nanoparticles in a Pickering stabilization approach.^29^ This method also increases the permeability of the interface. Various nanoparticle types have been employed for this purpose, *e.g.* poly(dopamine) particles,^30^ liposomes,^31^ microgels,^32^ latex particles,^33,34^ inorganic particles,^35–37^ or poly(lactic acid) platelets.^38^ A recent development is the stabilization *via* fatty acid bilayers, which, in contrast, significantly decreases interfacial permeability.^39^ Various potential applications for w/w emulsions are discussed. For example, Hao and coworkers employed w/w emulsions for biolubrication in animal models for the development of osteoarthritis treatments.^28^ In cosmetics, w/w emulsions are used in the formation of microbeads.^40^ Also for the use in food w/w emulsions are discussed frequently, which essentially enables fat-free emulsions that have similar properties to commonly used oil-containing emulsions^41,42^ as well as sprayable gels for food preservation.^43^ Although uses in food and cosmetics require adherence to various regulations, w/w emulsions can be formed from approved food-grade components, simplifying access to potential applications.^44–46^

ATPSs and w/w emulsions offer an interesting avenue for stimulus-responsive behaviour, achieved either through stimulus responsive polymers^47,48^ or through exploiting intrinsic features of their phase diagrams. For example, the shift of the binodal with temperature can be exploited to move between a one-phase and a two-phase system.^49^ As the two-phase systems are only stable at elevated concentrations, dilution can induce the transition from a two-phase to a one-phase region by crossing the binodal, thereby breaking an ATPS or demulsifying a w/w emulsion.^30^ This is a simple way to turn aqueous multi-phase systems one-phasic. Another approach is the use of stimulus-responsive stabilisation in w/w emulsions, *e.g.* by temperature^50,51^ or pH changes.^52,53^ As w/w emulsions are commonly stabilized by nanoparticles, the reversible stimulus-induced formation of nanoparticles or nanoparticles featuring stimulus-response can be exploited to adjust the properties of w/w emulsions. For example, w/w emulsions can be turned on or off^50^ as well as polymer accumulation adjusted by changes in wettability.^32^ Degradation introduces another avenue to control ATPS and w/w emulsion properties. The change in molecular weight/polymer content *via* degradation leads to a movement of the system state in the phase diagram and ultimately below the binodal to form a one-phase system. As such, degradation is affecting ATPSs and w/w emulsions on an angle that is succinct from previous approaches.

The present study investigates the disruption of ATPSs and w/w emulsions by orthogonal degradation of two polymers, focusing for the first time on systems formed from polyphosphoesters and Dex (Scheme 1). Orthogonality in degradation triggers will enable the design of complex aqueous multi-phase systems in the future, *e.g.* by using more than two polymers and combination with other stimuli. The present ATPS was designed using polymers that are both degradable. Dex is a biobased polysaccharide which degrades enzymatically by dextranase. PPEs are a class of polymers based on phosphoric or phosphonic acid, which give access to well-defined and water-soluble polymers susceptible to aqueous hydrolysis,^54,55^ while being stable in the presence of dextranase.^49,56^ As such, their hydrolytic degradation can be exploited as a stimulus to break the respective ATPS or w/w emulsion. ATPS formation was characterized by constructing a two-phase diagram, while degradation was studied using NMR spectroscopy. Emulsion formation and degradation were studied *via* optical microscopy. This new development in ATPSs and w/w emulsions introduces degradation as a novel stimulus for controlling the state of the aqueous multi-phase systems.

**Scheme 1:**
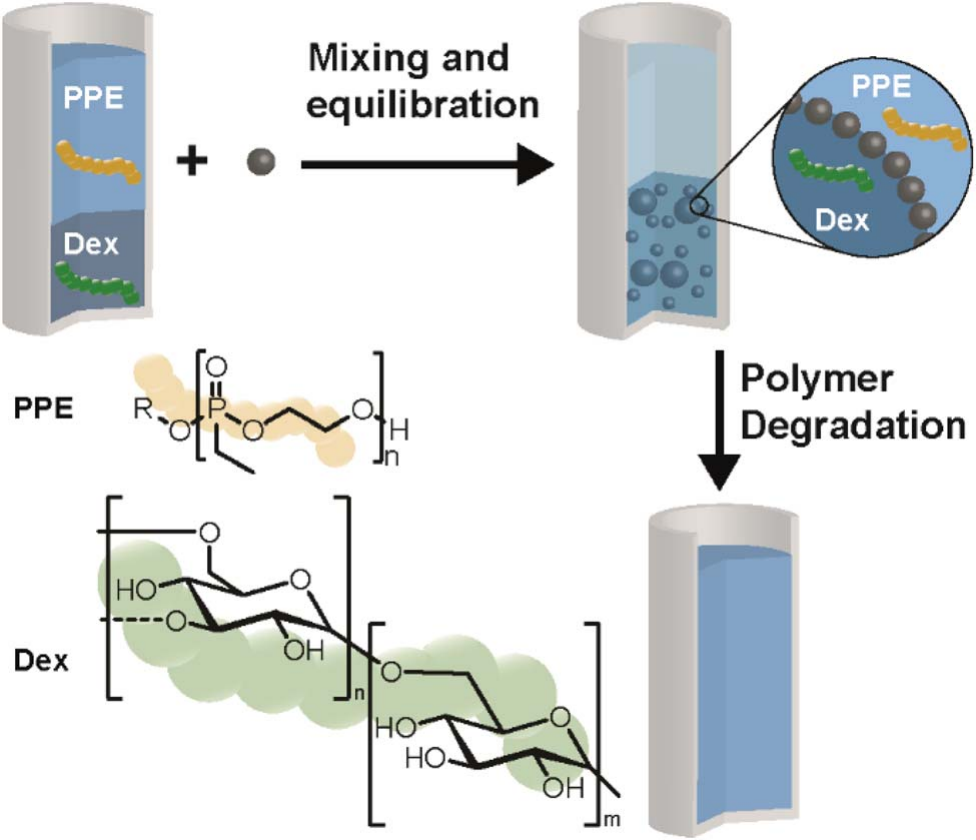
Overview of an ATPS from poly(ethylene ethyl phosphonate) (PPE) and dextran (Dex), w/w emulsion formation as well as selective degradation leading to destabilization of the emulsion.

In order to study ATPS formation, a hydrophilic polyphosphoester was synthesized, namely poly(ethyl ethylene phosphonate) (PPE), which is well known for its high water-solubility and similar properties, like antifouling or low protein adsorption.^57–59^ The organocatalytic ring-opening polymerization of 2-ethyl-2-oxo-1,3,2-dioxaphospholane was initiated by an approximate 8-mer of oligo(ethylene glycol) and catalyzed by 1,8-diazabicyclo[5.4.0]undec-7-ene (DBU) in dichloromethane.^60^ Oligo(ethylene glycol) was chosen as the initiator to improve solubility in the beginning of the reaction and allow easy determination of molar mass. Intentionally, hydrophobic initiators (*e.g.* like benzene-derivatives, which we used in previous PPE-studies) were not used to prevent any unwanted aggregation. The obtained PPE had a monomodal molecular weight distribution with a molecular weight of 14 000 g mol^−1^ and *Đ* of 1.03 (Fig. S1). The structure was confirmed *via*^1^H and ^31^P NMR spectroscopy (Fig. S2 and S3, respectively). The commercially sourced polysaccharide Dex with a molecular weight of 40 000 g mol^−1^ was used to form the two-phase system.

Both polymers were combined at elevated concentration to form an ATPS, which developed rapidly and showed clear, easily distinguishable phase separation. An initial 20 wt% total polymer concentration was used, showing phase separation with varying polymer ratios ranging from PPE/Dex 1 : 9 to 9 : 1 (Fig. 1a). In the resulting ATPS, the PPE-enriched phase forms the upper layer while the PPE depleted (and Dex-enriched) phase sits at the bottom. Next, these two-phase mixtures were diluted until only one phase was observed to enable the construction of a phase diagram with a binodal. The phase diagram features a rather large one-phase region, roughly between 14 wt% PPE and 16 wt% Dex content with an almost linear binodal (Fig. 1b). Consequently, the minimum total polymer concentration required for an ATPS is approximately 15 wt%.

**Fig. 1:**
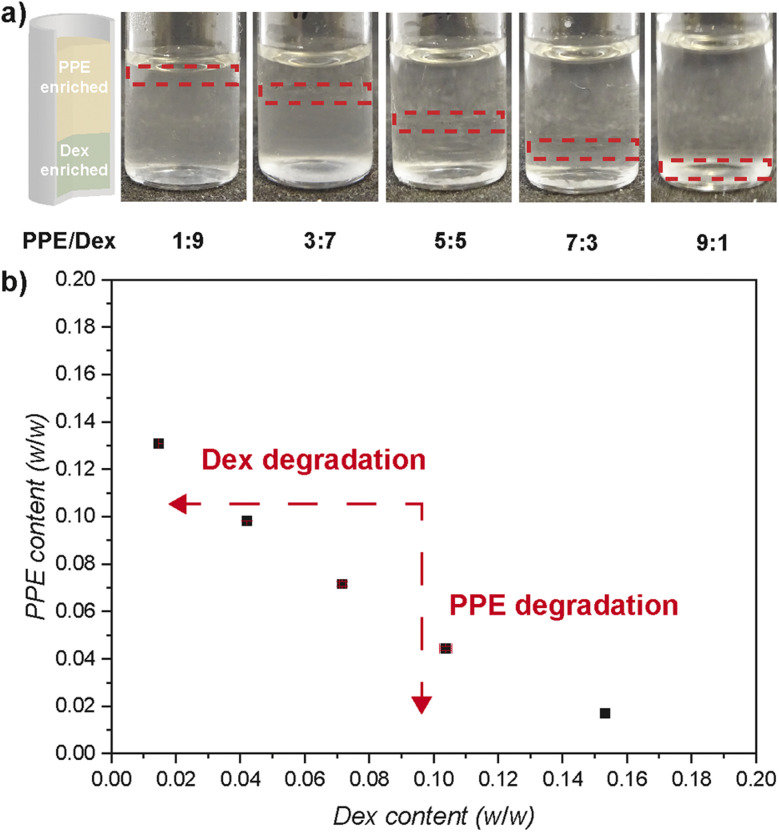
ATPS from PPE and Dex: (a) ATPS composed of PPE and Dex at various ratios (from left to right PPE/Dex: 2 wt%/18 wt%, 6 wt%/14 w%, 10 wt%/10 wt%, 14 wt%/6 wt%, and 18 wt%/2 wt%) and (b) phase diagram of the ATPS for PPE and Dex (black squares for the binodal) with red arrows indicating the effect of polymer degradation.

With the knowledge of ATPS formation and the phase diagram, w/w emulsion formation was investigated with a total polymer concentration of 20 wt%. As mentioned earlier, a Pickering stabilization approach is commonly employed for such emulsions. Hence, we used Ludox silica nanoparticles with a diameter of 22 nm and negative charge as stabilizers. One advantage of these particles is their transparent dispersions, which simplifies optical imaging. Two polymer ratios were studied: PPE/Dex 7 : 3 and PPE/Dex 3 : 7. According to the phase diagram, two polymer concentrations of PPE/Dex 14 wt%/6 wt% and PPE/Dex 6 wt%/14 wt% were selected, while the stabilizer concentration was fixed at 2 wt%. All components were mixed by vortexing, followed by ultrasound and manual shaking. After 24 h, phase separation was observed due to settling of the emulsion (Fig. S4). Optical microscopy revealed that the top phase contained only minor amounts of droplets (Fig. S5), whereas the bottom phase exhibited significant droplet presence in bright-field images, confirming emulsion formation (Fig. 2). To determine polymer enrichment in the emulsion, fluorescence microscopy was done using fluorescein-labelled PPE (FITC-PPE) (synthesized by chain-end modification with fluorescein-isothiocyanate) and commercially sourced fluorescein-labelled Dex (FITC-Dex). For the PPE/Dex 7 : 3 mixture, green fluorescence was observed in the continuous phase with FITC-PPE and in the droplets with FITC-Dex, consistent with the expectation that the polymer in excess preferentially partitions into the phase with larger volume. In the opposite case for PPE/Dex 3 : 7, fluorescence indicated a PPE enrichment inside of the droplets and Dex enrichment in the continuous phase. A broad droplet size dispersity was observed in the w/w emulsions, *i.e.* a mean diameter of 37.1 (±25.4) µm for PPE/Dex 7 : 3 and a mean diameter of 8.8 (±10.0) µm for PPE/Dex 3 : 7 (Fig. S6). The broad distribution is likely due to the preparation method, which was not optimized herein. Control over droplet size and dispersity might be improved through optimization of the dispersion process, such as use of microfluidics, homogenization or systematic variation of vortex and ultrasound parameters.

**Fig. 2:**
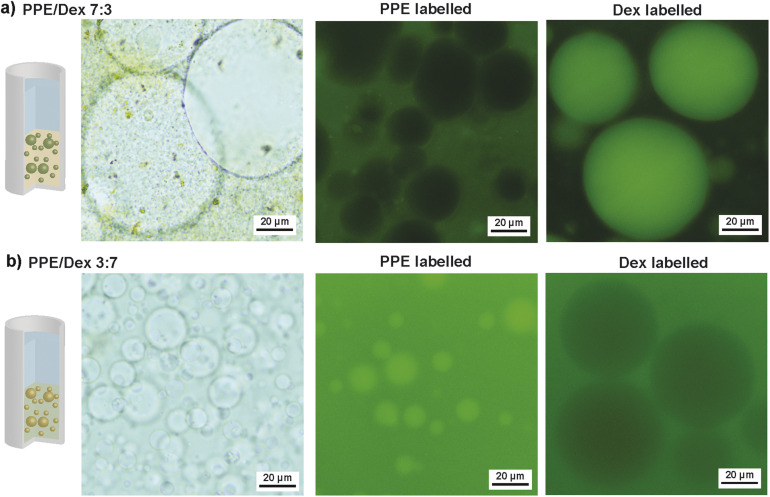
Optical and fluorescence microscopy of w/w emulsions in the bottom phase formed from PPE and Dex (left – bright field, middle – PPE FITC labelled, and right – Dex FITC labelled): (a) PPE/Dex/silica NPs 14 wt%/6 wt%/2 wt% and (b) PPE/Dex/silica NPs 6 wt%/14 wt%/2 wt%.

The PPE/Dex ATPS and w/w emulsion were specifically designed for their orthogonal degradability, allowing a selective destabilization of the hetero-phase system. The shift from an ATPS to a single-phase system and demulsification in the case of a w/w emulsions, can be explained with the thermodynamics of two-phase formation.^61^ On one hand, degradation leads to a reduced volume fraction of the degraded polymer. On the other hand, the position of the binodal depends on the molecular weight with a shift towards higher weight percentages for lower molecular weight. Both effects contribute to movement to the single-phase state eventually, *i.e.* lower volume fraction of one polymer type and reduced molecular weight. Therefore, we investigated the disruption of the ATPS *via* degradation stimuli. An ATPS containing PPE/Dex 10 wt%/10 wt% was formed, and the effect on the ATPS by degradation of each polymer type and degradation kinetics were studied individually. The PPE hydrolysis was triggered by the addition of NaOH solution (to a final NaOH concentration of 0.72 M), which induced rapid degradation of the polymer backbone in less than 1 minute,^62^ evidenced by the transition from two phases to one phase (Fig. 3a). Degradation was further confirmed by ^1^H and ^31^P NMR spectroscopy (Fig. 3b and S7). The methylene resonances of the phosphonic acid diester in the backbone at *ca.* 4.3 ppm disappeared and the resonances of the ethyl side-chain at 1.9 (methylene) and 1.2 ppm (methyl) shifted upfield after phosphoester cleavage. Also, the ^31^P NMR shows the hydrolysis by a shift to lower ppm (38.6 ppm to 31.2 ppm) as reported previously.^62^ The ^1^H NMR of Dex remained unaffected under these conditions as also shown in a control reaction making use of Dex and an aqueous NaOH solution (Fig. S8).

**Fig. 3:**
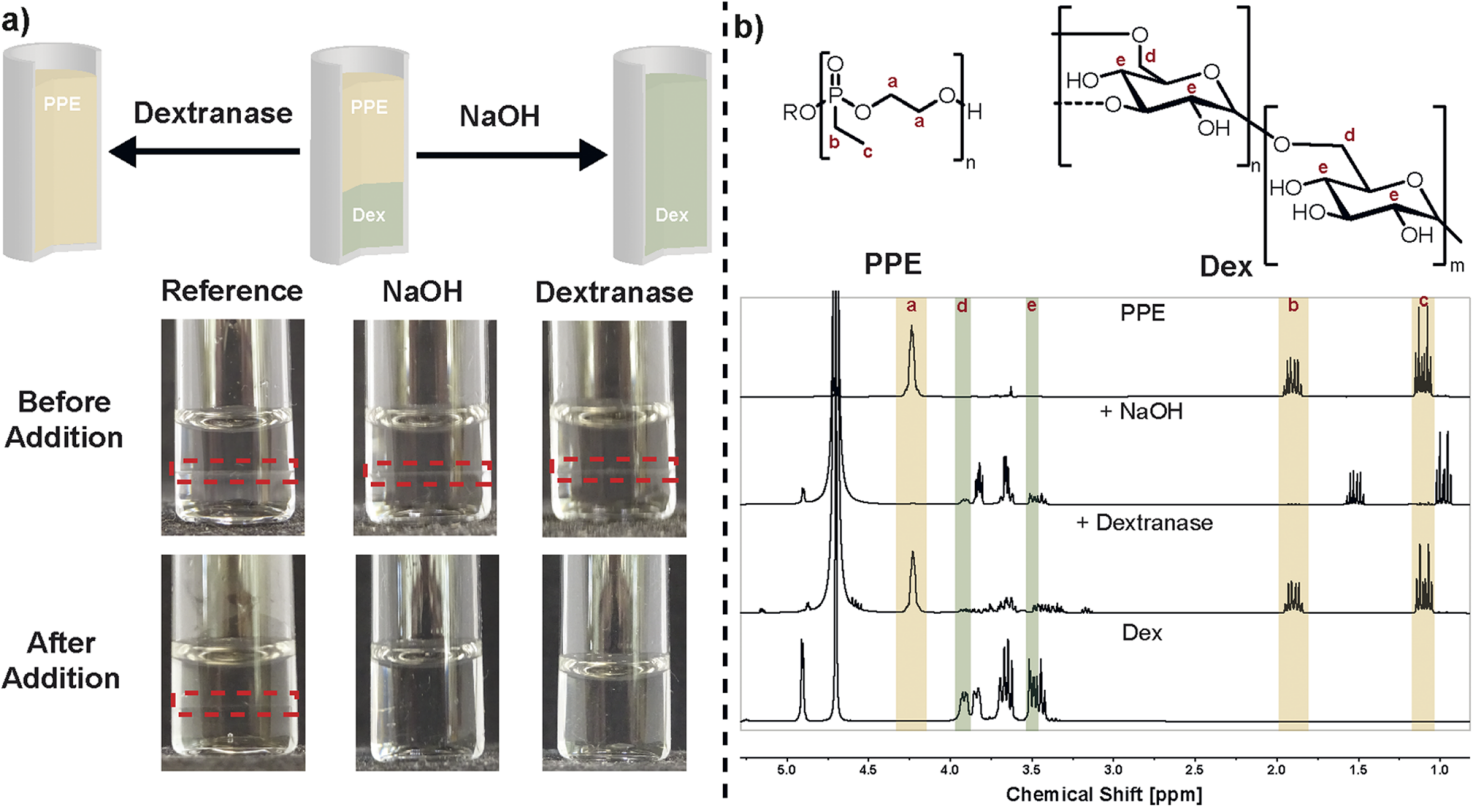
Degradation of ATPS (PPE/Dex 10 wt%/10 wt%): (a) photographs of ATPS before and after addition of reagent (left – reference after addition of deionized water (same dilution as for NaOH addition) and heating to 37 °C for 1 h, middle – after addition of aqueous NaOH solution (final concentration of 0.72 M) and right – after addition of dextranase and heating at 37 °C for 1 h). (b) ^1^H NMR spectra of homopolymer references and ATPS after degradation measured in D_2_O.

As an orthogonal trigger to destabilize the w/w emulsion, Dex was degraded enzymatically^63^ by adding dextranase and incubating at 37 °C for 1 h. An endodextranase isolated from *Penicillium* sp. was employed, which initially cleaves α − 1 → 6 glycosidic linkages to yield isomaltose as the major product, which is ultimately cleaved to glucose. Post-incubation, phase separation disappeared, resulting in a homogeneous solution (Fig. 3a). ^1^H NMR analysis confirmed degradation of Dex (Fig. 3b) by the shift of protons on C-4 and C-6 of the saccharide repeating units at 3.5 ppm and 3.9 ppm indicating the release of glucose, respectively. Under these conditions, PPE remained unaffected, as evidenced by ^1^H and ^31^P NMR signals with no significant shifts in the backbone, side-group of phosphorus peaks. This was also shown by a control reaction making use of PPE and dextranase only (Fig. S9). Diffusion-ordered spectroscopy (DOSY) further supported these findings, showing decreased diffusion coefficients for the non-degraded polymer (Fig. S10). To rule out effects of dilution and temperature on the state of the ATPS, a reference sample was prepared by diluting the PPE/Dex 10 wt%/10 wt% system with the same volume of water used for PPE degradation. After dilution an ATPS was still present. Additionally, incubating at 37 °C for 1 h without dextranase addition did not disrupt the two-phase system.

In order to gain a deeper insight into polymer degradation, reaction kinetics were studied. The kinetics of PPE hydrolysis were assessed in both a single-component aqueous solution (one-phase, 10 wt% PPE concentration) and in the 10/10 wt% ATPS by monitoring the change in the PPE CH_2_ resonance integral *ca* 1.9 ppm relative to the integral of the 1,3,5-trioxane internal standard in the ^1^H NMR spectrum over time (Fig. 4 and S11). A 3.25 M NaOH solution (final concentration of 0.68 M) was used instead of 4 M to prevent complete and rapid degradation of PPE and thereby enable kinetic analysis. Upon NaOH addition, hydrolysis of the phosphoesters occurred, reaching approximately 80% in the single-phase system and approximately 83% for the ATPS including a shift to a one phase system; however, no further degradation was observed with increasing reaction time. These results are consistent with previously reported NaOH-mediated PPE degradation kinetics^64^ and suggest that the presence of the Dex phase has minimal effect on the NaOH-mediated degradation process. Furthermore, the pH of the ATPS before and after addition of the NaOH solution was measured using a pH meter (Fig. S12). Before NaOH addition the pH was mildly acidic at a measured pH = 3.15, likely due to the presence of residual phosphoric acid due to slight PPE hydrolysis after standing in air over time. Immediately following NaOH addition, the pH rose to approximately pH = 12.5, and gradually dropped to a measured pH = 9.80, 40 minutes after addition (Fig. S12).

**Fig. 4:**
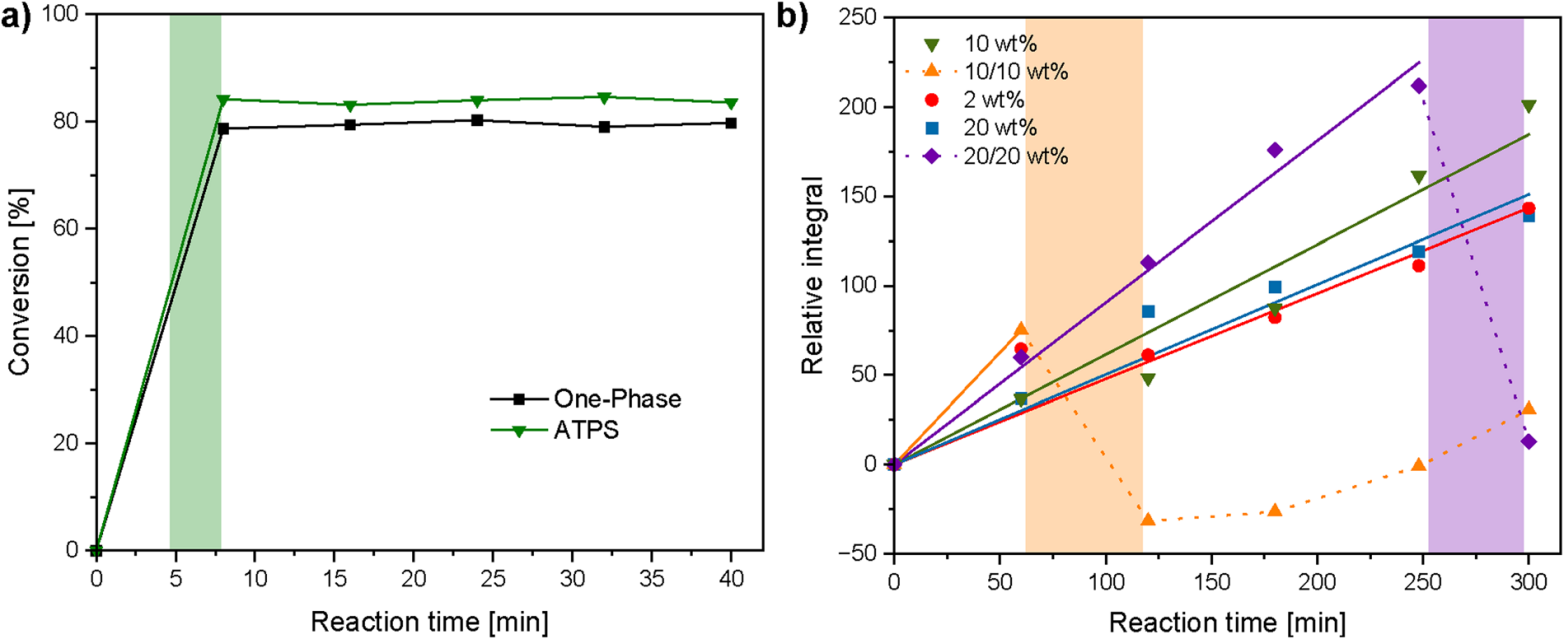
Degradation kinetics: (a) kinetics of NaOH-mediated degradation of PPE in water (black squares) and in the 10/10 wt% PPE/Dex two-phase system (green triangles, transition period indicated as a green area) and (b) kinetics of dextranase-mediated degradation of Dex in aqueous systems at 2, 10, and 20 wt% (red circles, green triangles, and blue squares, respectively), the 10/10 wt% PPE/Dex ATPSs (orange triangles, transition period indicated as an orange area) and the 20/20 wt% PPE/Dex ATPSs (purple diamonds, transition period indicated as a purple area). The dotted lines link the data points taken where the ATPS had broken and mixed into a single-phase.

The kinetics of dextranase-mediated Dex degradation were studied both as a single component aqueous solution (one-phase, Dex concentrations of 2 wt%, 10 wt% and 20 wt%) and in the ATPS (Dex/PPE concentration of 10/10 wt% as well as 20/20 wt%) by monitoring the relative growth of the isomaltose resonance (*ca.* 3.43 ppm) integral against the integral of a benzyl alcohol internal standard in the ^1^H NMR spectrum over time (Fig. 4, S13 and S14). In the one-phase systems, there was a linear growth of the residual integral over time (*R*^2^ = 0.98 for both 10 and 20 wt% systems), with the 10 wt% system having a slightly higher degradation rate compared to the 20 wt% system. The 10/10 wt% ATPS showed a higher initial relative integral compared to all one-phase systems, but had visually transitioned to a single phase by 120 minutes. This is reflected by the large decrease in the relative integral from 75 (*t* = 60 min) to −31 (*t* = 120 min) as a consequence of dilution upon phase mixing, which then increased linearly to 31 (*t* = 300 min). Increasing polymer concentrations to 20/20 wt% slowed the transition from two distinct phases to a single phase which was instead visually apparent by 300 minutes, and reflected by a large decrease in the relative integral from 212 (*t* = 248 min) to 13 (*t* = 300 min). The 20/20 wt% ATPS retained a linear increased degradation rate until 240 minutes (*R*^2^ = 0.996), but was notably higher compared to all one-phase systems. We wondered if high substrate or product concentrations may inhibit dextranase activity and thereby limit degradation rates in the one-phase systems, as reported previously for dextranase activity at high substrate concentrations.^65^ However, a similar degradation rate to the 10 and 20 wt% one-phase systems was observed when kinetic measurements were conducted at a significantly lower Dex concentration (2 wt%). Overall, the presence of the PPE phase appears to accelerate dextranase-mediated Dex degradation. A similar enhancement in dextranase activity has been previously observed in a poly(ethylene glycol)/Dex ATPS,^66^ and was attributed to dextranase compartmentalisation which overcomes substrate inhibition.

Breaking of the ATPS from PPE and Dex *via* polymer degradation motivated the subsequent investigation of demulsification through similar degradation stimuli (Fig. 5). As before, PPE/Dex 7 : 3 and PPE/Dex 3 : 7 were studied as polymer ratios (PPE/Dex 14 wt%/6 wt% and PPE/Dex 6 wt%/14 wt%) with a stabilizer concentration of 2 wt%. The emulsions were prepared as before and equilibrated for 24 h, after which settling was again observed.

**Fig. 5:**
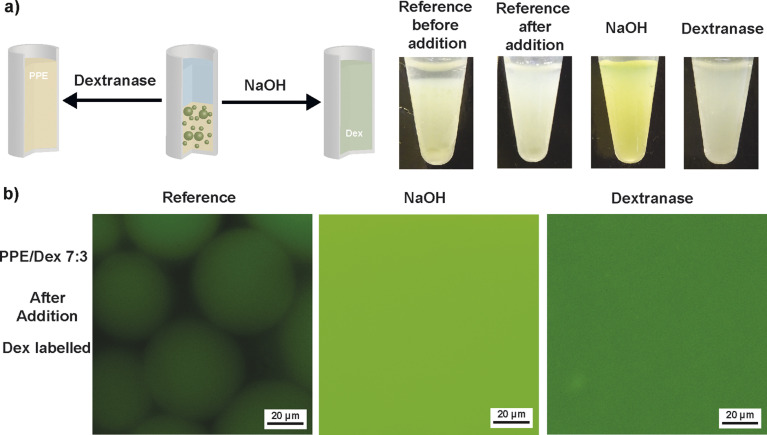
Degradation of w/w emulsions (PPE/Dex/silica NPs 14 wt%/6 wt%/2 wt%): (a) photographs of w/w emulsions before and after addition of reagent (from left to right: reference before addition of deionized water, reference after addition of deionized water (same dilution as for addition of aqueous NaOH solution) and heating to 37 °C for 1 h, after addition of aqueous NaOH solution (final concentration of 0.23 M) and after addition of dextranase and heating to 37 °C for 1 h. (b) Fluorescence microscopy images of w/w emulsions with FITC labelled Dex (from left to right: reference after addition of deionized water (same dilution as for addition of aqueous NaOH solution) and heating to 37 °C for 1 h, after addition of aqueous NaOH solution (final concentration of 0.23 M) and after addition of dextranase and heating to 37 °C for 1 h.

For the PPE/Dex 7 : 3 system, addition of aqueous NaOH solution to a final concentration of 0.23 M led to breaking of emulsion droplets after 1 minute. Bright-field microscopy revealed a crowded dispersion of undefined polymer aggregates rather than emulsion droplets (Fig. S15). Fluorescence microscopy using FITC-Dex confirmed the loss of the emulsion droplet structure, as fluorescence was evenly distributed throughout the sample. A control sample of the silica stabilizers under the conditions for PPE degradation, *i.e.* addition of aqueous NaOH solution (final concentration of 0.72 M), was investigated as well to exclude demulsification through particle degradation. Dynamic light scattering and zeta potential measurements showed only minor differences before and after base addition (Fig. S16 and Table S1), which suggests preservation of stabilizer particles under degradation conditions.

Similarly, degradation of Dex was probed. Enzymatic degradation by dextranase incubation at 37 °C for 1 h also led to breaking of emulsion droplets, as evidenced by homogeneous fluorescence. Reference samples treated with water instead of NaOH and heating retained intact emulsions in both bright-field and fluorescence microscopy before and after treatment. Analogous observations were made for the PPE/Dex ratio 3 : 7 as well (Fig. S17). The uniform fluorescence throughout the degraded samples suggests these systems hold promise for applications in encapsulation, delivery and controlled release triggered by polymer degradation within an all-aqueous environment.

In conclusion, we present the formation of a new ATPS utilizing dextran and a water-soluble polyphosphoester. Two-phase formation occurs readily, enabling construction of a phase diagram to allow precise adjustment of polymer concentrations. Subsequently, w/w emulsions were formed with polymer ratios of PPE/Dex 7 : 3 or 3 : 7 making use of silica nanoparticles as stabilizers. While emulsification was achieved using simple methods, phase settling led to a supernatant nearly free of droplets. Fluorescence microscopy confirmed partitioning of the polymer constituents into droplets or continuous phase, consistent with the majority polymer enriching in the continuous phase and the minority polymer localizing within droplets. The system was designed to investigate the effect of polymer degradation on ATPSs and w/w emulsions. Hence, degradation of PPE and Dex was triggered by aqueous NaOH and dextranase, respectively, resulting in rapid transitions from two phases to a single phase, as observed visually and by NMR spectroscopy. Kinetic analysis showed similar PPE degradation in water and the ATPS, whereas Dex degradation was accelerated in the ATPS. This degradation stimulus was also effective in demulsifying w/w emulsions. Overall, these findings provide new opportunities to manipulate ATPSs and w/w emulsions not only through their polymer components but also *via* controlled degradation. This strategy opens promising avenues for developing novel all-aqueous systems that feature a degradation stimulus.

## Author contributions

The manuscript was written through contributions of all authors. All authors have given approval to the final version of the manuscript. Conceptualization: BS and FRW. Data curation: BS, JMC, and RE. Funding acquisition: BS and FR. Investigation: BS, JMC, RE, and NB. Writing – original draft: BS. Writing – review & editing: BS, JMC, FRW, NB, and RE.

## Conflicts of interest

There are no conflicts of interest to declare.

## Supplementary Material

SC-OLF-D5SC09406B-s001

## Data Availability

The data supporting this article have been included as part of the supplementary information (SI). Supplementary information: Fig. S1: SEC data; Fig. S2, S3, S7–S11, S13, and S14: NMR spectra; Fig. S4, S5 and S6 (droplet sizes), S15, and S17: microscopy data; Fig. S12: pH data; Fig. S16, Table S1: DLS data; and further experimental details. Experimental procedures and analytical data on the utilised polymers; analytical data on the multi-phase systems; analytical data on the w/w emulsions. See DOI: https://doi.org/10.1039/d5sc09406b.
