# Supplementary material for: Degradable polyphosphoester-based aqueous two-phase systems and water-in-water emulsions
Source: Chem Sci. 2026 Jan 9;17(10):5220–8. doi: 10.1039/d5sc09406b (PMC12817469; doi:10.1039/d5sc09406b)
Supplement: SC-017-D5SC09406B-s001 [file SC-017-D5SC09406B-s001.pdf]

Supplementary Information (SI) for Chemical Science.
This journal is © The Royal Society of Chemistry 2026

Supporting Information

Degradable polyphosphoester-based aqueous two-phase systems and water-in-water emulsions

Jordan A. J. McCone,[†] *Ramon ten Elshof,*[‡] *Niamh Bayliss,*[†] *Frederik R. Wurm,*[‡,]* *Bernhard V. K. J. Schmidt*[†,]*

[†] School of Chemistry, University of Glasgow, Glasgow G12 8QQ, UK

[‡] Sustainable Polymer Chemistry, Department of Molecules and Materials, MESA+ Institute for Nanotechnology, Faculty of Science and Technology, University of Twente, P.O. Box 217, 7500 AE, Enschede, the Netherlands.

Email:

bernhard.schmidt@glasgow.ac.uk

frederik.wurm@utwente.nl

Experimental Part:

Materials

Dextran40 (Dex, 40 kg mol^{-1}, TCI), dextranase (from *Penicillium sp.*, 100-250 units mg^{-1} protein, Sigma Aldrich), dibutyltin dilaurate (95%, Sigma Aldrich), dimethylsulfoxide (DMSO, dry over molecular sieves, Acros), Fluorescein isothiocyanate (FITC, Sigma Aldrich), Fluorescein isothiocyanate dextran (FITC-Dex, Sigma Aldrich), LUDOX® TM-50 colloidal silica 50 wt% suspension in water (22 nm diameter, Sigma Aldrich) and sodium hydroxide (≥97.0%, Sigma Aldrich) were obtained from commercial sources. Deionized water was obtained from an ELGA Purelab Option system. 2-Ethyl-2-oxo-1,3,2-dioxaphospholane was synthesized according to the two-step procedure described by Wolf *et al.*[1]

Synthesis of poly(ethylene ethyl phosphonate) (PPE)

Polymerization was performed according to modified literature protocols.[1] The respective monomers were weighed in a flame-dried Schlenk-tube, dissolved in dry benzene and dried three times by lyophilization. The monomer was dissolved in dry dichloromethane to a total concentration of 4 mol L^{-1}. A stock solution of initiator oligoethylene glycol in dry dichloromethane was prepared with a concentration of 0.2 mol L^{-1} and the calculated amount was added to the monomer solution via Hamilton® syringe. A stock solution of DBU in dry dichloromethane was prepared with a concentration of 0.2 mol L^{-1}. The monomer solution and the catalyst solution were cooled to 0 °C.

The polymerization was initiated by the addition of the calculated volume of catalyst solution containing 3.0 equivalents of DBU in respect to the initiator's OH groups. Polymerization was terminated by the rapid addition of an excess of formic acid dissolved in dichloromethane with a

concentration of 20 mg mL^{-1}. The colorless, amorphous polymer was purified by precipitation into cold diethyl ether, dialyzed against water over night (MWCO 3500 Da) and lyophilized (frozen in liquid nitrogen and dried at 10^{-1} mbar). Yield: approx. 90%. ^{1}H NMR ($CDCl_3$, ppm): δ = 4.26–4.10 (m, backbone –CH_2-), 3.55 (s, initiator CH_2-O-), 1.82–1.68 (m, side-chain P-CH_2-), 1.17–1.05 (m, side-chain –CH_3). ^{31}P NMR ($CDCl_3$, ppm): δ = 35.2.

Synthesis of fluorescein-labelled PPE (FITC-PPE)

According to the literature,[2] PPE (100 mg) was dissolved in dry DMSO (1 mL). FITC (13.8 mg) was then added, followed by dibutyltin dilaurate (4.5 mg). The reaction mixture was heated to 95 °C for 2 h. Subsequently, the labeled polymer was dialyzed (3.5 kDa MWCO) against deionized water over three days. Finally, the solution was lyophilized (frozen in liquid nitrogen and dried at 10^{-1} mbar) to yield the product as yellow oil (98 mg).

Preparation of ATPS and phase diagram

PPE (100 mg) and Dex (100 mg) were dissolved in deionized water (800 mg) to obtain a 10.0 wt% / 10.0 wt% mixture. The mixture was left over night to reach complete dissolution and two-phase formation. Subsequently, the solution was diluted with 100 mg of deionized water, shaken by hand for 30 seconds, equilibrated at ambient temperature for 30 minutes in order to phase separate and the solution state investigated by the presence or absence of a phase boundary. The process was repeated until the phase boundary was no longer observed. The average weight concentration between the last addition and the second to last addition was recorded as the data point for the binodal curve. All other concentration combinations (2 wt%/18 wt%, 6 wt%14 wt%, 14 wt%/6 wt%, 18 wt%/2 wt%) were analyzed in a similar way. Each dilution sequence was repeated three times and the data point for the binodal curve was obtained from the average.

Exemplary PPE hydrolysis in ATPS

PPE (62 mg) and Dex (62 mg) were dissolved in deionized water (496 mg) to obtain a 10.0 wt% / 10.0 wt% mixture. After two phase formation, aqueous NaOH solution (4 M, 110 µL) was added and the transition to one phase observed within 1 min.

Exemplary Dex degradation procedure in ATPS

PPE (62 mg) and Dex (62 mg) were dissolved in deionized water (496 mg) to obtain a 10.0 wt% / 10.0 wt% mixture. After two phase formation, dextranase (1 mg) was added and the solution heated to 37 °C for 1 h, when one phase was observed.

Degradation reference for ATPS

PPE (62 mg) and Dex (62 mg) were dissolved in deionized water (496 mg) to obtain a 10.0 wt% / 10.0 wt% mixture. After two phase formation, 110 µL deionized water was added and two phases were observed. Next, the solution heated to 37 °C for 1 h and two phases were observed.

Exemplary preparation of w/w emulsions using LUDOX nanoparticles

PPE (29 mg), FITC-PPE (1 mg) and Dex (70 mg) were dissolved in deionized water (379 mg). Ludox nanoparticle dispersion (14 µL) was added to generate a 2 wt% dispersion and a PPE/Dex 6/14 wt% solution. The mixture was subjected to vortex for 1 min, ultrasound (Bandelin Sonorex RK100, 35 kHz, 80 W) for 1 min, shaken by hand for 1 min, and analyzed after 24 h by optical and fluorescence microscopy.

Exemplary PPE degradation procedure in w/w emulsion

PPE (21 mg), FITC-Dex (1 mg) and Dex (48 mg) were dissolved in deionized water (266 mg). Ludox nanoparticle dispersion (10 µL) was added to generate a 2 wt% dispersion and a PPE/Dex 6/14 wt% solution. The mixture was subjected to vortex for 1 min, ultrasound (Bandelin Sonorex RK100, 35 kHz, 80 W) for 1 min and shaken by hand for 1 min. After two phase formation, aqueous NaOH solution (4 M, 17 µL) was added and the transition to one phase observed within 1 min. The degraded emulsion was analyzed by optical and fluorescence microscopy.

Exemplary Dex degradation procedure in w/w emulsion

PPE (21 mg), FITC-Dex (1 mg) and Dex (48 mg) were dissolved in deionized water (266 mg). Ludox nanoparticle dispersion (10 µL) was added to generate a 2 wt% dispersion and a PPE/Dex 6/14 wt% solution. The mixture was subjected to vortex for 1 min, ultrasound for 1 min and shaken by hand for 1 min. After two phase formation, dextranase (0.3 mg) was added and the emulsion heated to 37 °C for 1 h. The degraded emulsion was analyzed by optical and fluorescence microscopy.

Degradation reference for w/w emulsion

PPE (21 mg), FITC-Dex (1 mg) and Dex (48 mg) were dissolved in deionized water (266 mg). Ludox nanoparticle dispersion (10 µL) was added to generate a 2 wt% dispersion and a PPE/Dex 6/14 wt% solution. The mixture was subjected vortex for 1 min, ultrasound (Bandelin Sonorex RK100, 35 kHz, 80 W) for 1 min, shaken by hand for 1 min, and analyzed after 24 h by optical and fluorescence microscopy. After two phase formation, deionized water (17 µL) was added and

the transition to one phase observed within 1 min. Next, the emulsion was heated to 37 °C for 1 h. The emulsion was analyzed by optical and fluorescence microscopy.

Control study of Dex degradation with NaOH

Dex (29 mg) was suspended in deionized water (256 mg). The solution was subjected to vortex for 1 min, then shaken by hand for 1 min. NaOH (114 μL of a 3.25 M solution) was then added and the mixture was lightly shaken and left to sit for 24 h.

Procedure for studying Dex degradation kinetics in water (10 wt% Dex)

Dex (52 mg) and benzyl alcohol (6 mg) were suspended in deionized water (390 mg). The mixture was subjected to vortex for 1 min, ultrasound for 1 min and then shaken by hand for 1 min. A 57 μL aliquot was taken from the mixture, diluted with D_2O (450 μL) and analyzed by ^{1}H NMR for the t = 0 reference point, using the Ar-H environments at 4.77–7.36 ppm in benzyl alcohol as the internal standard, setting the integral to 1000. Dextranase (0.021 mg in 41.3 μL of deionized water, approx. 0.0045 wt%) was then added and the mixture was continuously shaken to give a final Dex concentration of 10 wt%. A 57 μL aliquot was taken every 60 mins, diluted with D_2O (0.45 mL), and rapidly analyzed by ^{1}H NMR, monitoring Dex degradation by the growth of a multiplet at 3.30–3.39 ppm by integration.

Procedure for studying Dex degradation kinetics in w/w emulsion (10/10 wt% Dex/PPE)

PPE (80 mg), FITC-PPE (2 mg), benzyl alcohol (7.4 mg) and Dex (82 mg) were suspended in deionized water (580 mg). The mixture was subjected to vortex for 1 min and ultrasound for 1 min, then shaken by hand for 1 min. After equilibrating for 24 h, a 57 μL aliquot was taken from the

bottom phase, diluted with D_2O (0.45 mL) and analyzed by ^1H NMR for the t = 0 reference point, using the Ar-H environment at 7.77–7.36 ppm in benzyl alcohol as the internal standard, setting the integral to 1000. Dextranase (0.035 mg in 70 μL of deionized water, approx. 0.0045 wt%) was then added and the mixture was lightly shaken to give a final PPE/Dex concentration of 10/10 wt%. A 57 μL aliquot was taken from the bottom phase every 60 mins, diluted with D_2O (0.45 mL), and rapidly analyzed by ^1H NMR, monitoring Dex degradation by the growth of a multiplet at 3.30–3.39 ppm by integration.

Procedure for studying Dex degradation kinetics in water (20 wt% Dex)

Dex (100 mg) and benzyl alcohol (10 μL) were suspended in deionized water (390 mg). The mixture was subjected to vortex for 1 min and ultrasound for 1 min, then shaken by hand for 1 min. A 57 μL aliquot was taken from the mixture, diluted with D_2O (450 μL) and analyzed by ^1H NMR for the t = 0 reference point, using the Ar-H environments at 4.77–7.36 ppm in benzyl alcohol as the internal standard, setting the integral to 1000. Dextranase (0.05 mg in 100 μL deionized water, approx. 0.009 wt%) was then added and the mixture was continuously shaken to give a final Dex concentration of 20 wt%. A 57 μL aliquot was taken every 60 mins, diluted with D_2O (0.45 mL), and rapidly analyzed by ^1H NMR, monitoring Dex degradation by the growth of a multiplet at 3.30–3.39 ppm by integration.

Procedure for studying Dex degradation kinetics in water (2 wt% Dex)

Dex (100 mg) and benzyl alcohol (10 μL) were suspended in D_2O (3.99 g). The mixture was subjected to vortex for 1 min and ultrasound for 1 min, then shaken by hand for 1 min. After equilibrating for 24 h, a 570 μL aliquot was taken from the reaction and analyzed by ^1H NMR for

the t = 0 reference point, using the Ar-H environments at 4.77–7.36 ppm in benzyl alcohol as the internal standard, setting the integral to 1000. Dextranase (0.05 mg in 100 μL deionized water, approx. 0.001 wt%) was then added and the mixture was continuously shaken to give a final Dex concentration of 2 wt%. A 570 μL aliquot was taken every 60 mins and rapidly analyzed by ^{1}H NMR, monitoring Dex degradation by the growth of a multiplet at 3.30–3.39 ppm by integration.

Procedure for studying Dex degradation kinetics in w/w emulsion (20/20 wt% Dex/PPE)

PPE (200 mg), FITC-PPE (2 mg), benzyl alcohol (20 μL) and Dex (200 mg) were suspended in deionized water (580 mg). The mixture was subjected to vortex for 1 min and ultrasound for 1 min, then shaken by hand for 1 min. After equilibrating for 24 h, a 57 μL aliquot was taken from the bottom phase, diluted with D_2O (0.45 mL) and analyzed by ^{1}H NMR for the t = 0 reference point, using the Ar-H environment at 7.77–7.36 ppm in benzyl alcohol as the internal standard, setting the integral to 1000. Dextranase (0.1 mg in 200 μL deionized water, approx. 0.009 wt%) was then added and the mixture was lightly shaken to give a final PPE/Dex concentration of 20/20 wt%. A 57 μL aliquot was taken from the bottom phase every 60 mins, diluted with D_2O (0.45 mL), and rapidly analyzed by ^{1}H NMR, monitoring Dex degradation by the growth of a multiplet at 3.30–3.39 ppm by integration.

Control study of PPE degradation with dextranase

PPE (50 mg) was suspended in deionized water (280 mg). The solution was subjected to vortex for 1 min and ultrasound for 1 min, then shaken by hand for 1 min. Dextranase (0.5 mg) was then added and the mixture was stirred for 24 h at 37 °C.

Procedure for studying PPE degradation kinetics in water

PPE (50 mg) and 1,3,5-trioxane (2.5 mg) was suspended in deionized water (400 mg). The mixture was subjected to vortex and ultrasound for 1 min, then shaken by hand for 1 min. After equilibrating for 24 h, a 31 μL aliquot was taken, diluted with D_2O (0.45 mL) and analyzed by ^1H NMR for the t = 0 reference point, using the CH_2 environment at 5.23 ppm in 1,3,5-trioxane as the internal standard. A solution of NaOH (98 μL of a 3.25 M solution) was then added and the mixture was lightly shaken. A 31 μL aliquot was taken every 8 mins, diluted with D_2O (0.45 mL), and analyzed by ^1H NMR, monitoring PPE degradation by integrating the triplet at 1.66–1.56 ppm.

Procedure for studying PPE degradation kinetics in ATPS

PPE (100 mg), FITC-PPE (2 mg), Dex (100 mg) and 1,3,5-trioxane (5 mg) was suspended in deionized water (800 mg). The mixture was subjected to vortex and ultrasound for 1 min, then shaken by hand for 1 min. After equilibrating for 24 h, a 40 μL aliquot was taken from the top phase, diluted with D_2O (0.45 mL) and analyzed by ^1H NMR for the t = 0 reference point, using the CH_2 environment at 5.23 ppm in 1,3,5-trioxane as the internal standard. A solution of NaOH (196 μL of a 3.25 M solution) was then added and the mixture was lightly shaken. A 40 μL aliquot was taken from the top phase every 8 mins, diluted with D_2O (0.45 mL), and analyzed by ^1H NMR, monitoring PPE degradation by integrating the triplet at 1.66–1.56 ppm.

Characterization Methods.

^1H NMR spectra were recorded in deuterium oxide (D_2O, Sigma Aldrich) at ambient temperature at 400 MHz with a Bruker Ascend400. ^{31}P-NMR spectra were recorded in deuterium oxide (D_2O, Aldrich) at ambient temperature at 162 MHz with a Bruker Ascend400. Diffusion ordered

spectroscopy (DOSY) was measured in DMSO-d_6 using the ledbpgp2s pulse sequence. Size exclusion chromatography (SEC) of PPE was conducted in DMF (Sigma Aldrich) with 0.1 M LiBr (Sigma Aldrich) at 50 °C using a column system with a PSS GRAM-30A and PSS GRAM-1000A column and Agilent 1260 Infinity II RID detector. The system was calibrated with poly(styrene) standards. Optical and fluorescence microscopy was performed using a Visiscope IT406 FL2 trinocular inverted microscope. The size of the emulsion droplets was determined over 100 droplets from optical microscopy images and averaged. The error is based on the standard deviation. Dynamic light scattering (DLS) was measured with an Anton Paar Litesizer 500. The pH of the Dex/PPE ATPS before and after treatment with NaOH solution was measured using the HI-9810382 Halo 2 pH meter, and universal indicator pH paper.

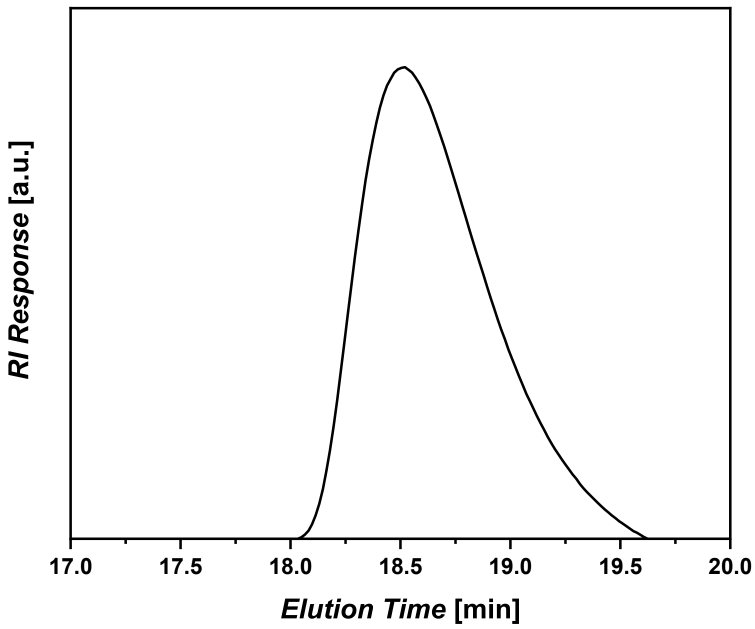

Figure S1. SEC elugram of PPE measured in DMF with 0.1 M LiBr.

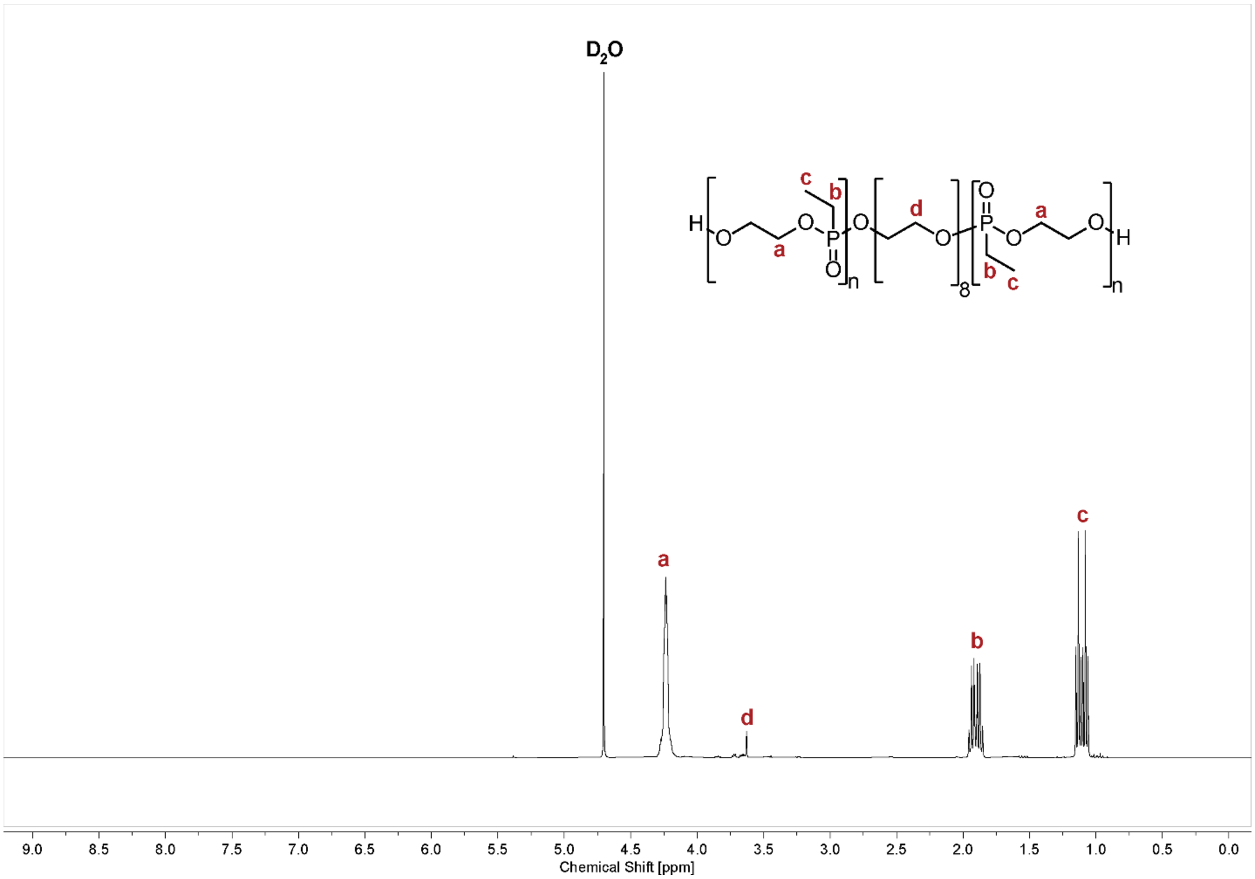

Figure S2. ^{1}H NMR spectrum of PPE measured in D_2O.

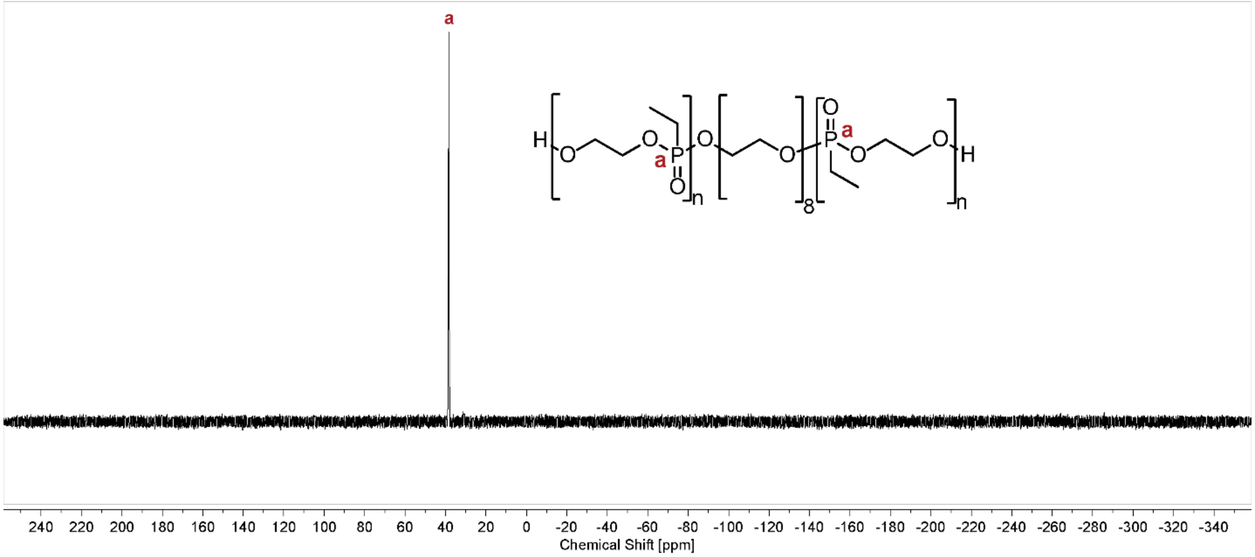

Figure S3. ^{31}P NMR spectrum of PPE measured in D_2O.

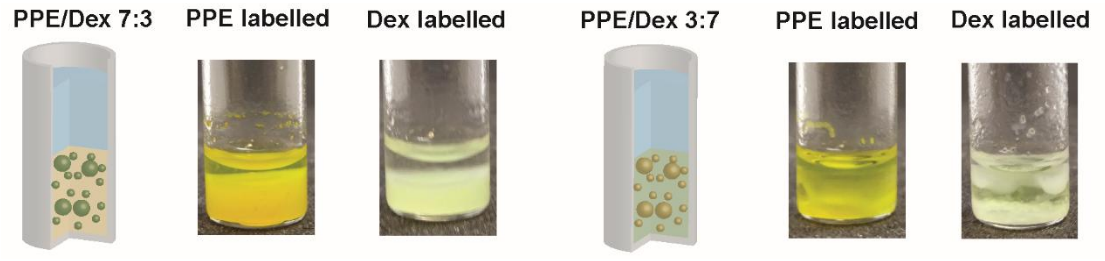

Figure S4. Photographs of w/w emulsions, left to right: PPE/Dex/Silica NPs 14 wt%/6 % / 2 wt% (PPE labelled), PPE/Dex/Silica NPs 14 wt%/ 6 wt% / 2 wt% (Dex labelled), PPE/Dex/Silica NPs 6 wt%/ 14 wt% / 2 wt% (PPE labelled) and PPE/Dex/Silica NPs 6 wt%/ 14 wt% / 2 wt% (Dex labelled).

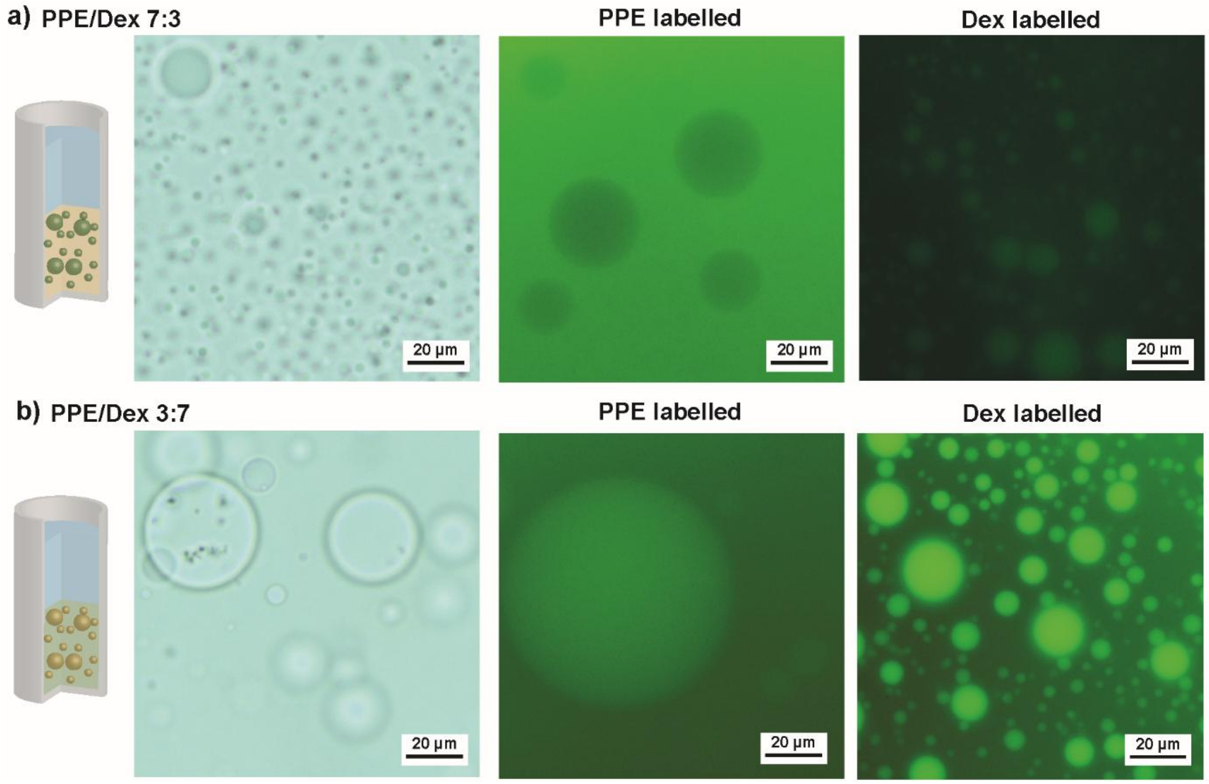

Figure S5. Optical microscopy of top phases in w/w emulsions samples formed from PPE and Dex (left bright-field, middle PPE FITC labelled, right Dex FITC labelled): a) PPE/Dex/Silica NPs 14 wt%/6 wt%/2 wt% and b) PPE/Dex/Silica NPs 6 wt%/14 wt%/2 wt%.

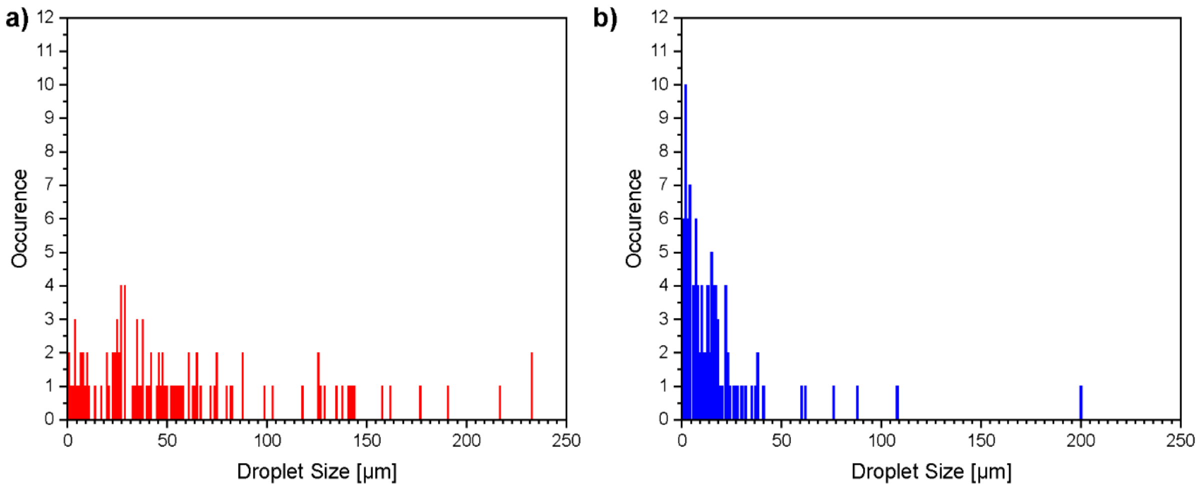

Figure S6. Droplet size distributions obtained from bottom phases via optical microscopy (calculated over a number of 100 droplets): a) PPE/Dex/Silica NPs 14 wt%/6 wt%/2 wt% and b) PPE/Dex/Silica NPs 6 wt%/14 wt%/2 wt%.

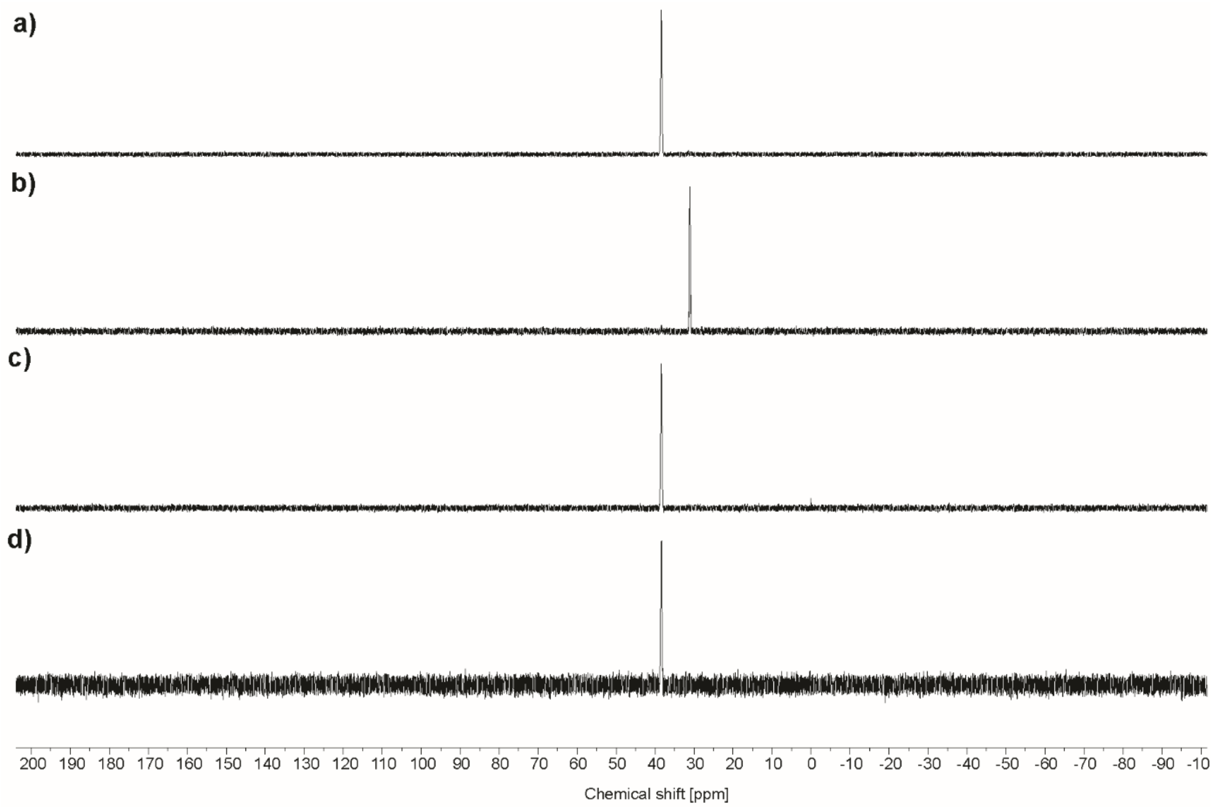

Figure S7. ^{31}P NMR spectra after degradation measured in D_2O: a) PPE before degradation, b) ATPS from PPE/Dex after addition of aqueous NaOH solution (final concentration of 0.72 M), c) PPE/Dex ATPS after addition of dextranase and d) PPE/Dex ATPS after dilution.

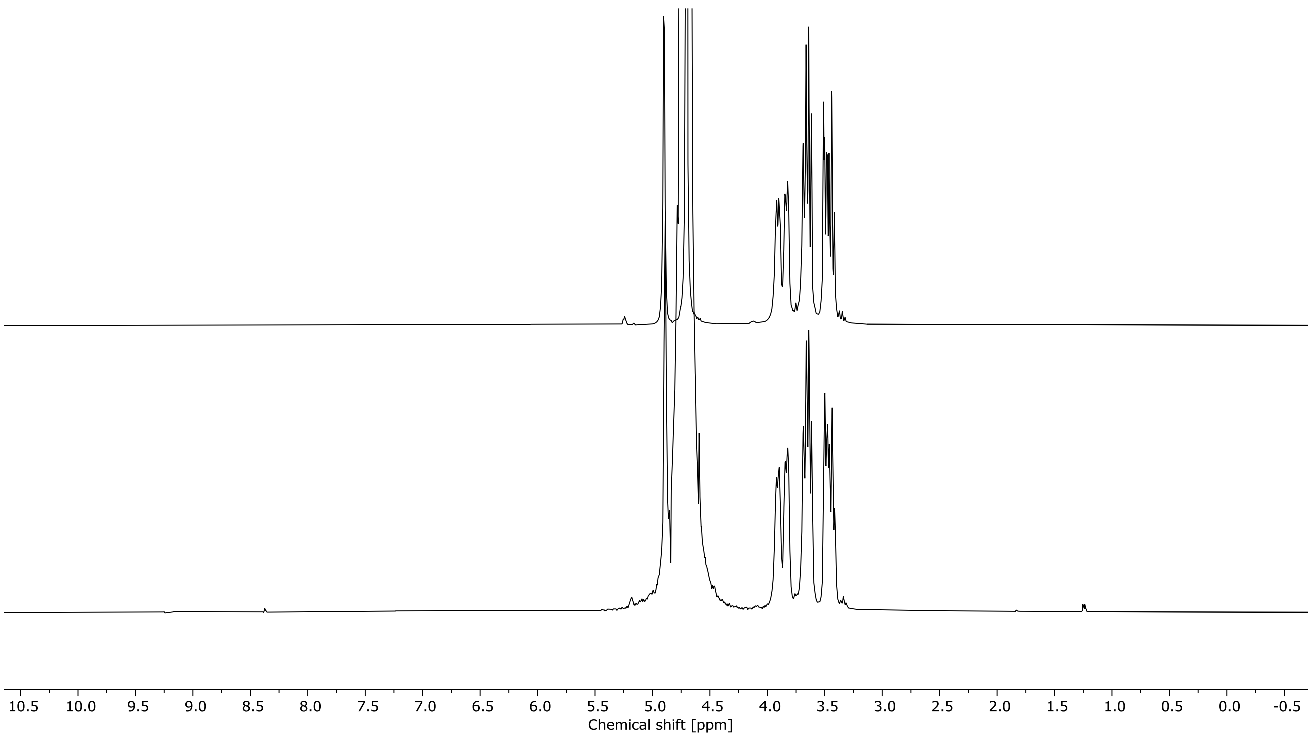

Figure S8. ^1H NMR spectra of Dex in D_2O before (top) and after (bottom) addition of aqueous NaOH solution (final concentration of 0.68 M) and incubating at ambient temperature for 24 h.

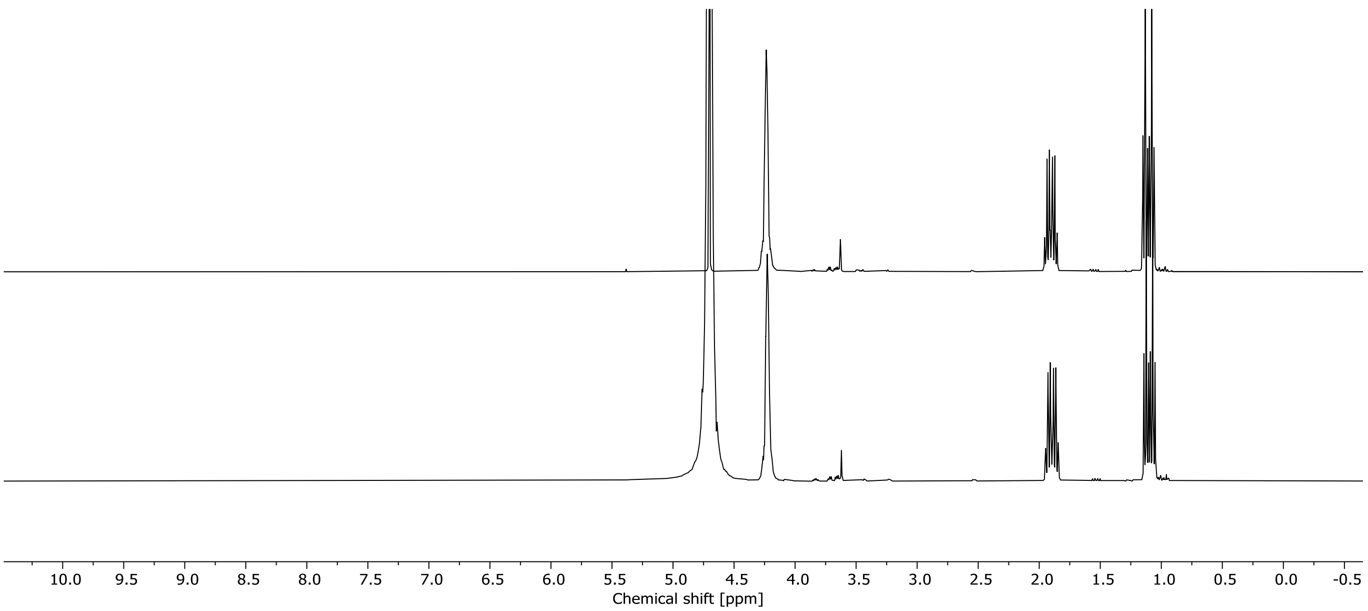

Figure S9. ^{1}H NMR spectra of PPE in D_2O before (top) and after (bottom) treatment with dextranase and incubation at 37 °C for 24 h.

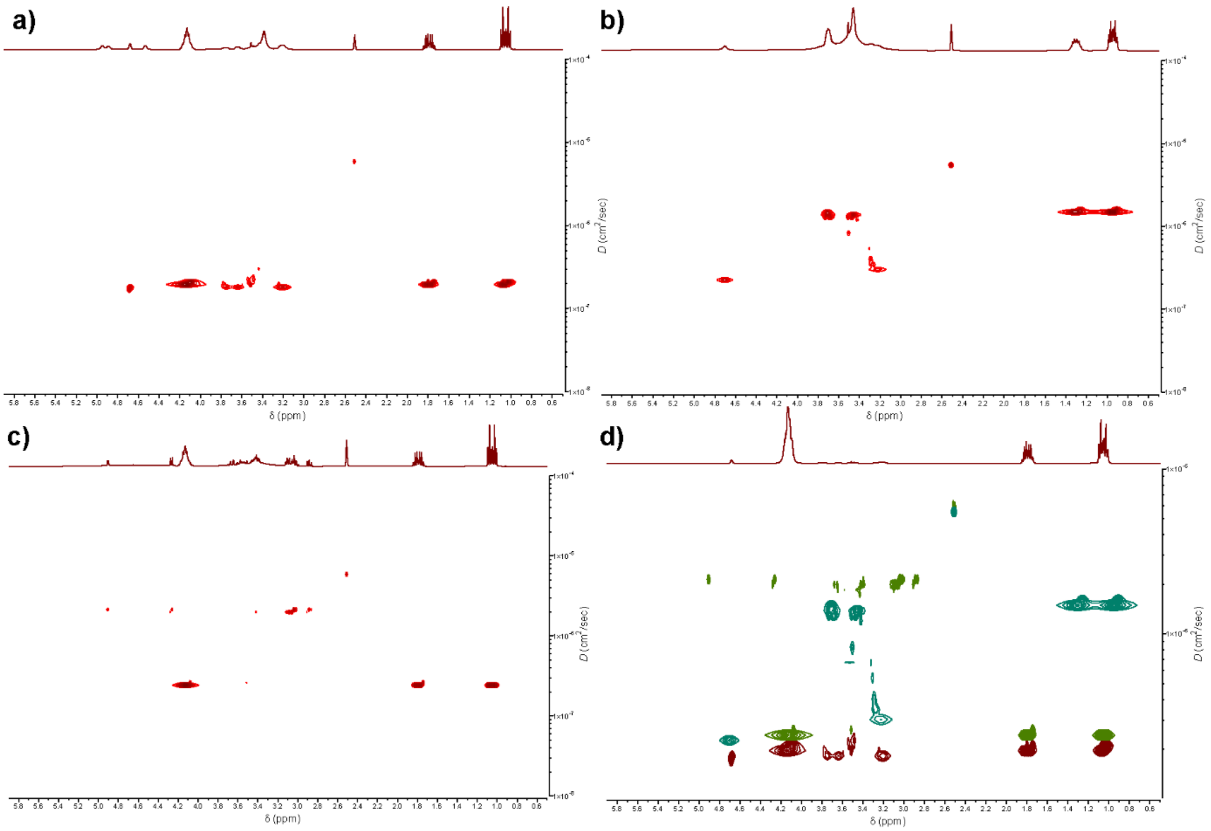

Figure S10. ^{1}H DOSY spectra after degradation measured in DMSO-d_6: a) ATPS from PPE/Dex before degradation, b) ATPS from PPE/Dex after addition of aqueous NaOH solution, c) PPE/Dex ATPS after addition of dextranase and d) overlay of all DOSY spectra (red: before degradation, turquoise: after addition of aqueous NaOH solution, green: after dextranase addition).

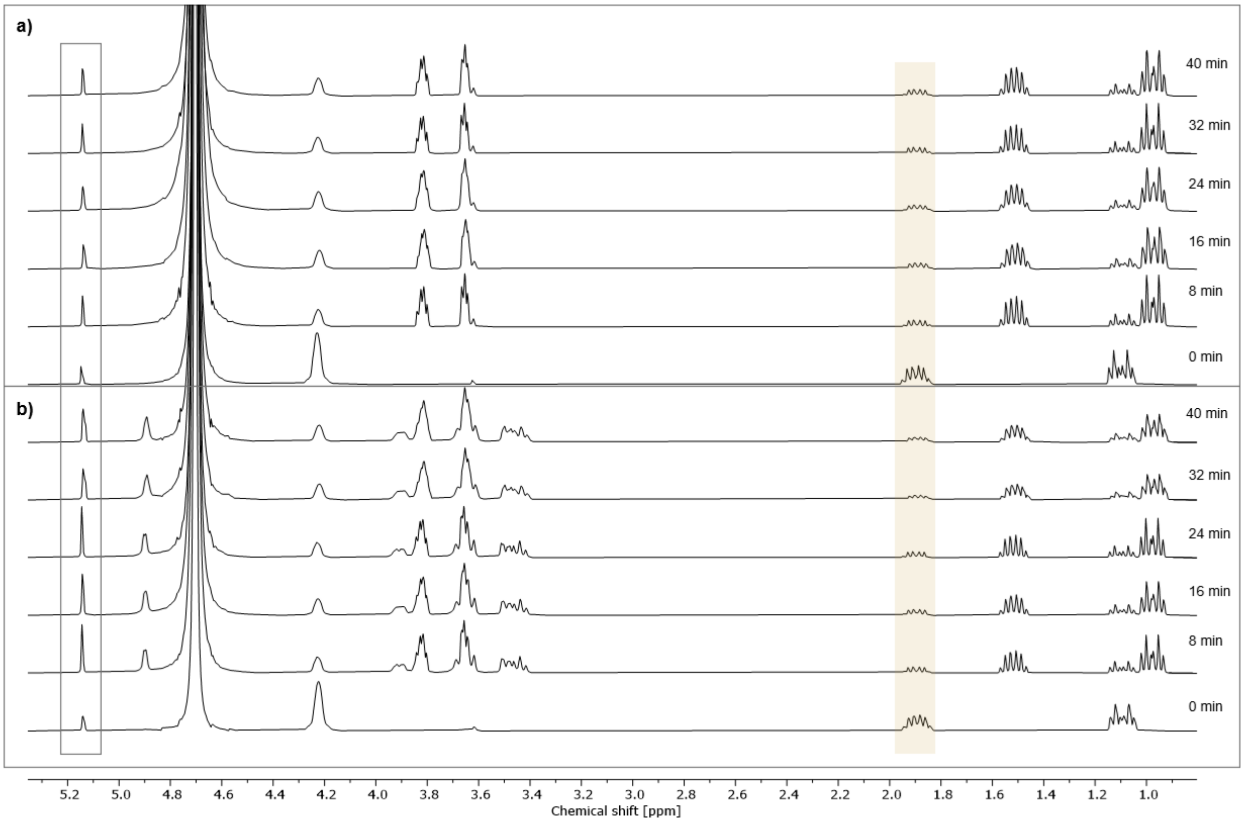

Figure S11. ^{1}H NMR spectra of PPE hydrolysis with NaOH measured in D_2O over time for determination of degradation kinetics: a) in the aqueous (one-phase) system, and b) in the ATPS. The resonance of the internal standard 1,3,5-trioxane is shown in the grey box, and the PPE resonance used to measure degradation is highlighted in the orange box.

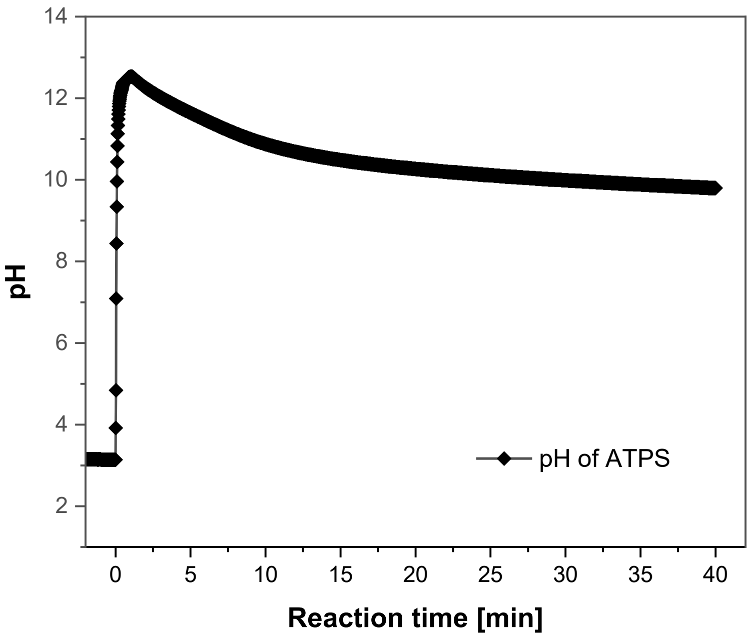

Figure S12. The change in pH of the ATPS (10/10 wt% PPE/Dex) over 40 minutes after addition of 3.25 M NaOH solution (final concentration of 0.68 M).

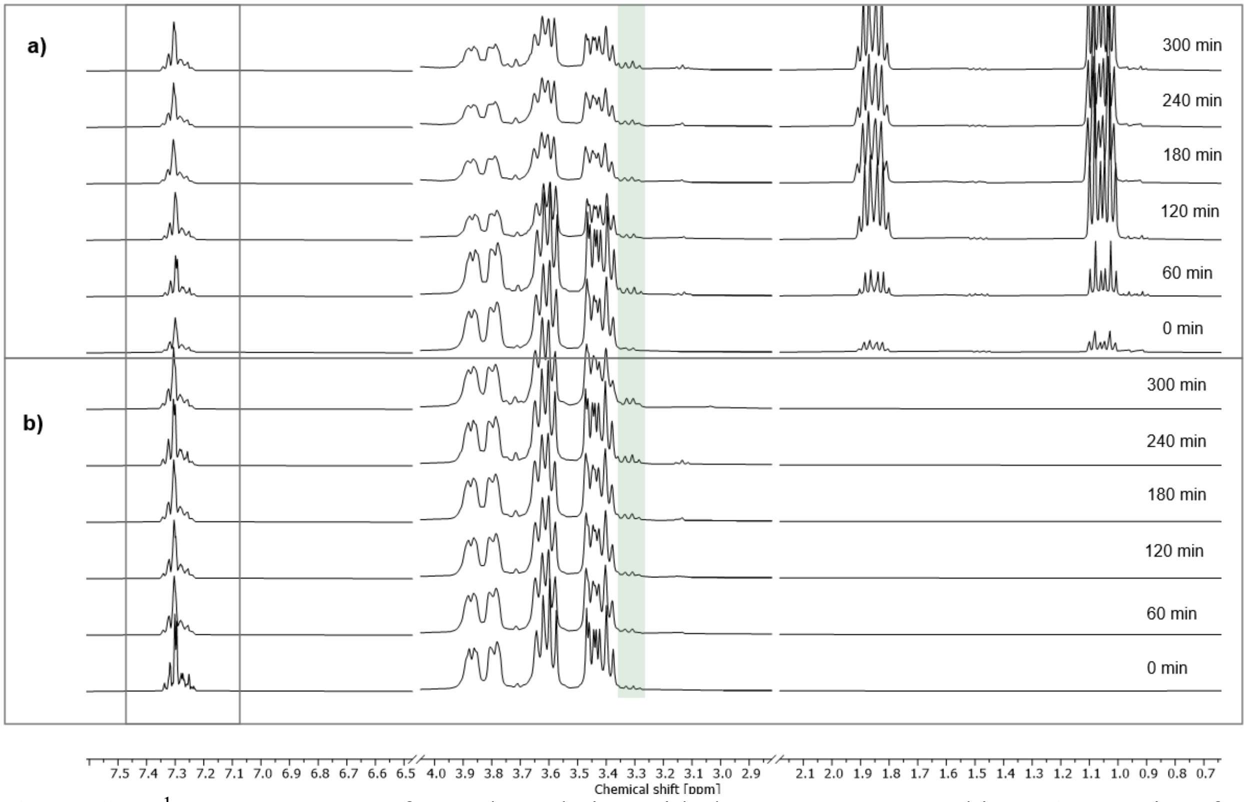

Figure S13. ^{1}H NMR spectra of Dex degradation with dextranase measured in D_2O over time for determination of degradation kinetics: a) in the ATPS with a Dex/PPE concentration of 10/10 wt%, b) in the aqueous (one-phase) system with a Dex concentration of 10 wt%. The resonance of the internal standard benzyl alcohol is shown in the grey box, and the Dex resonance used to measure degradation is highlighted in the green box.

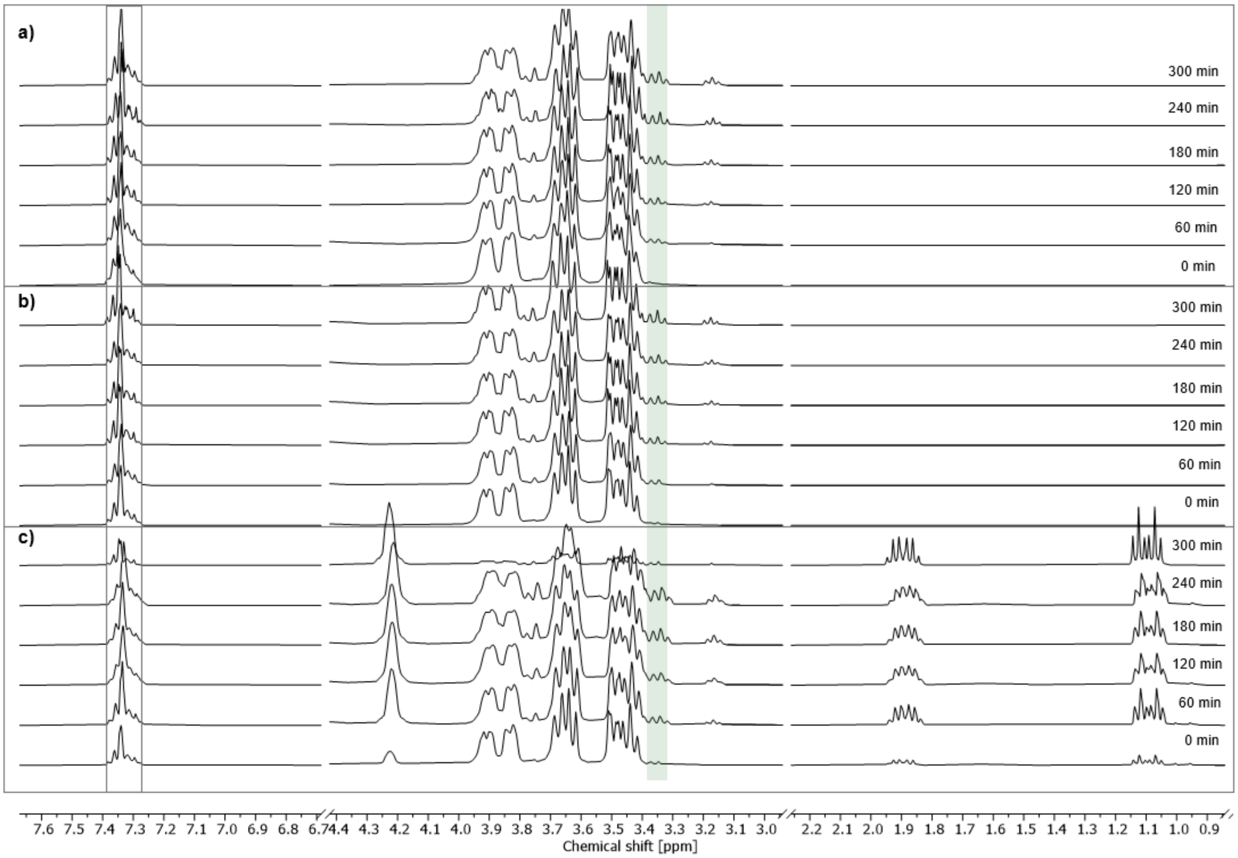

Figure S14. ^{1}H NMR spectra of Dex degradation with dextranase measured in D_2O over time for determination of degradation kinetics: a) in the aqueous (one-phase) system with a Dex concentration of 20 wt%, b) in the aqueous (one-phase) system with a Dex concentration of 2 wt%, c) in the 20/20 wt% ATPS. The resonance of the internal standard benzyl alcohol is shown in the grey box, and the Dex resonance used to measure degradation is highlighted in the green box.

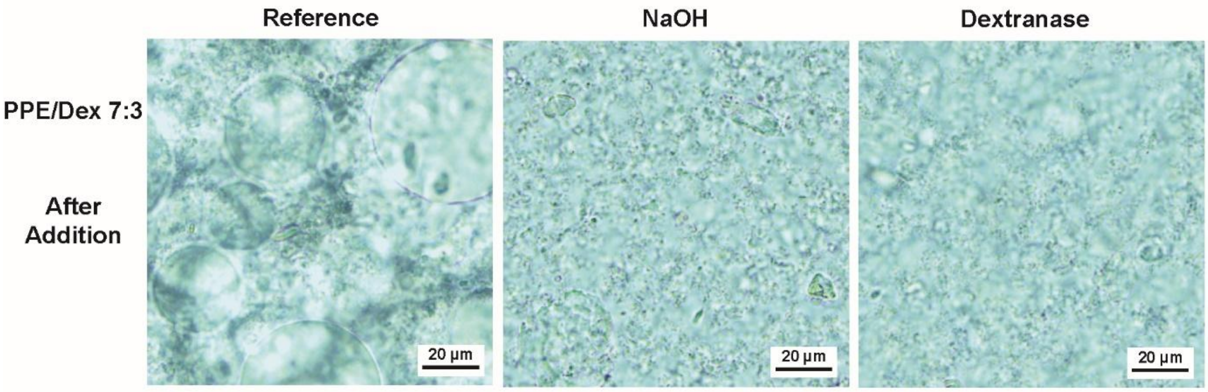

Figure S15. Degradation of w/w emulsions (PPE/Dex/Silica NPs 14 wt% / 6 wt% / 2 wt%): Bright-field microscopy images of w/w emulsions (from left to right: reference after addition of deionized water (same dilution as for addition of aqueous NaOH solution) and heating to 37 °C for 1 h, after addition of aqueous NaOH solution (final concentration of 0.23 M) and after addition of dextranase and heating to 37 °C for 1 h).

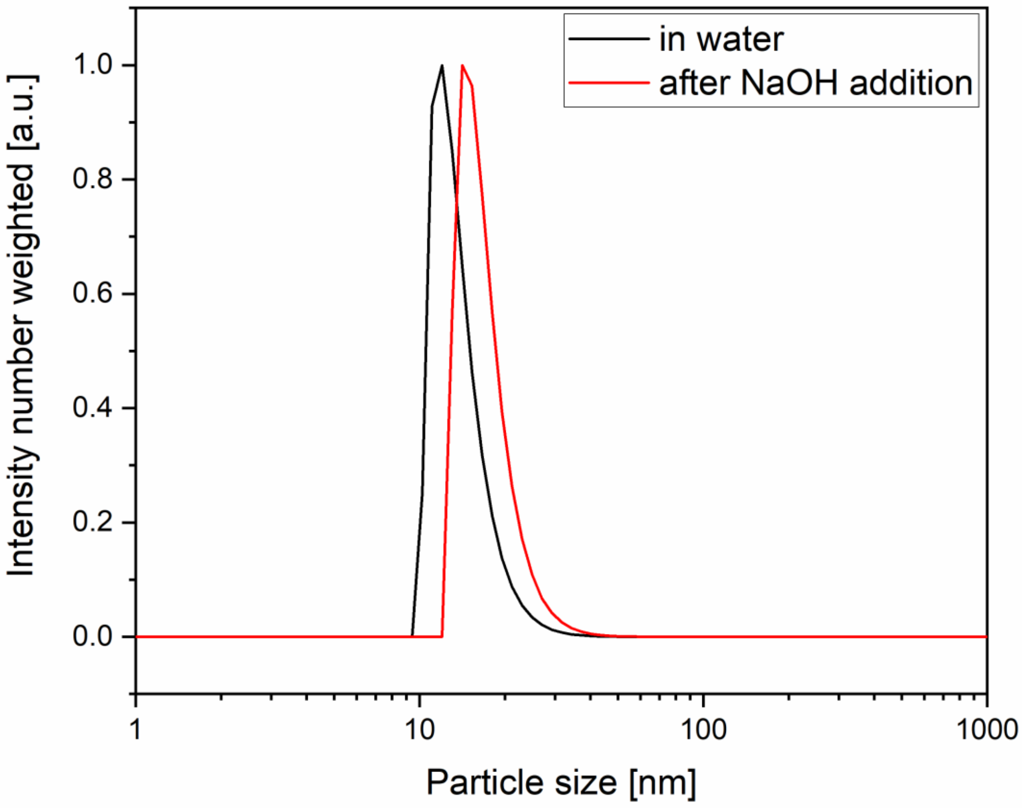

Figure S16. Comparison of number weighted particle size distribution of silica particles in water before (black curve) and after addition of aqueous NaOH solution (final concentration of 0.72 M) (red curve) at a concentration of 2 wt%.

Table S1. Number weighted particle sizes and zeta potentials of silica particles in water before (black curve, Figure S13) and after addition of aqueous NaOH solution (final concentration of 0.72 M) (red curve, Figure S13) at a concentration of 2 wt%.

**Sample**	**D_h [nm]**	**ζ-potential [mV]**
Silica particles in water	13.8 ± 2.5	-40.2 ± 1.4
Silica particles in 0.72 M NaOH	17.0± 2.8	-35.7 ± 1.4

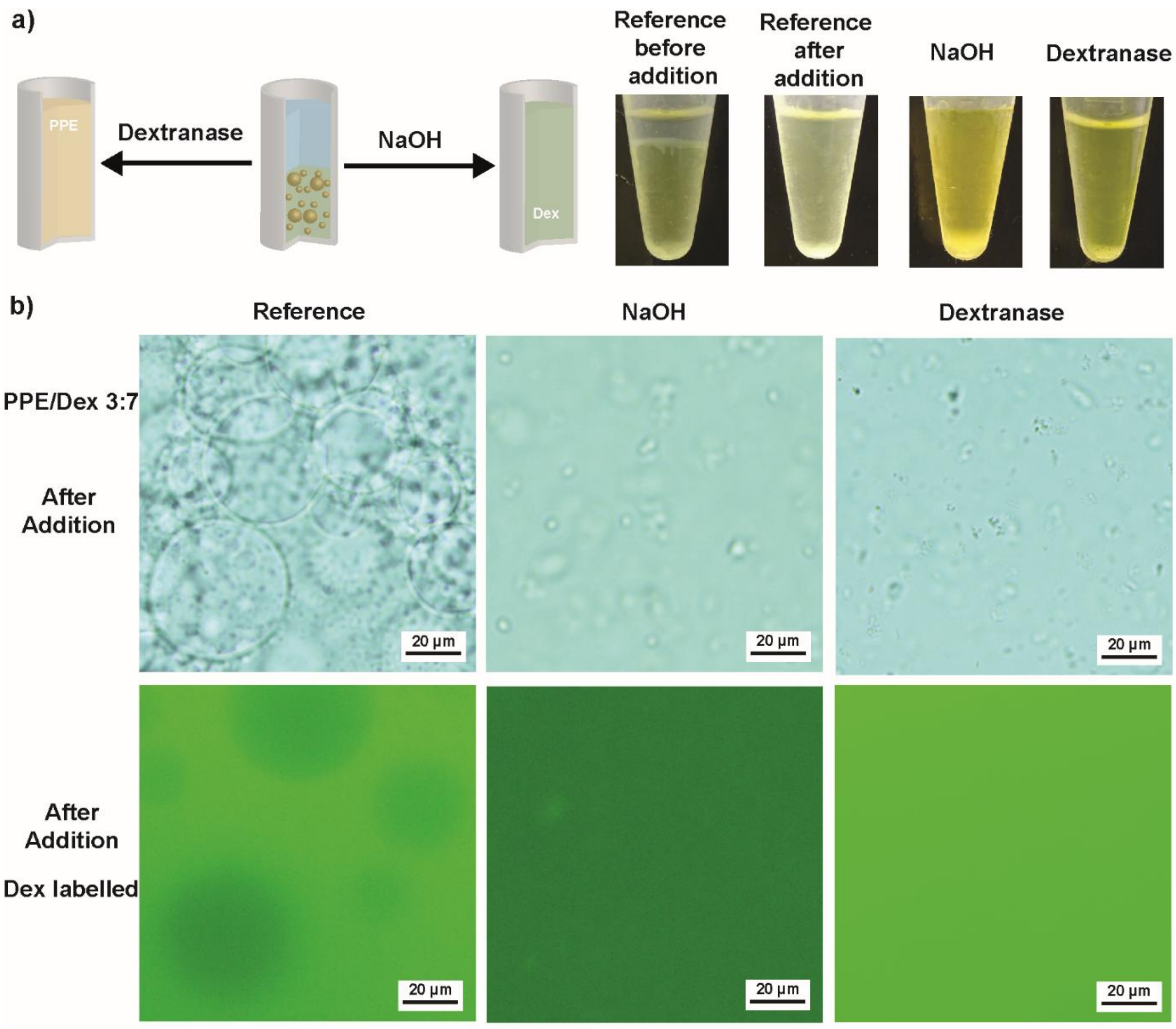

Figure S17. Degradation of w/w emulsions (PPE/Dex/Silica NPs 6 wt% / 14 wt% / 2 wt%): a) Photographs of w/w emulsions before and after addition of reagent (from left to right: reference before addition of deionized water, reference after addition of deionized water (same dilution as for addition of aqueous NaOH solution) and heating to 37 °C for 1 h, after addition of aqueous NaOH solution (final concentration of 0.23 M) and after addition of Dextranase and heating to 37 °C for 1 h). b) Bright-field microscopy images of w/w emulsions (from left to right: reference after addition of deionized water (same dilution as for addition of aqueous NaOH solution) and heating to 37 °C for 1 h, after addition of aqueous NaOH solution (final concentration of 0.23 M) and after

addition of dextranase and heating to 37 °C for 1 h). c) Fluorescence microscopy images of w/w emulsions with FITC labelled Dex (from left to right: reference after addition of deionized water (same dilution as for addition of aqueous NaOH solution) and heating to 37 °C for 1 h, after addition of aqueous NaOH solution (final concentration of 0.23 M) and after addition of dextranase and heating to 37 °C for 1 h).

REFERENCES

[1] Wolf, T.; Steinbach, T.; Wurm, F. R., *Macromolecules* **2015**, *48* (12), 3853-3863.

[2] Plucinski, A.; Schmidt, B. V. K. J., *Polym. Chem.* **2022,** *13* (28), 4170-4177.
